# Impairing committed cholesterol biosynthesis in white matter astrocytes, but not grey matter astrocytes, enhances in vitro myelination

**DOI:** 10.1111/jnc.15113

**Published:** 2020-07-31

**Authors:** Inge L. Werkman, Janine Kövilein, Jenny C. de Jonge, Wia Baron

**Affiliations:** ^1^ Biomedical Sciences of Cells & Systems section Molecular Neurobiology University of Groningen University Medical Center Groningen Groningen the Netherlands; ^2^ Present address: Department of Biology University of Virginia Charlottesville VA USA

**Keywords:** astrocytes, cholesterol, cytokines, myelin, oligodendrocyte

## Abstract

Remyelination is a regenerative process that is essential to recover saltatory conduction and to prevent neurodegeneration upon demyelination. The formation of new myelin involves the differentiation of oligodendrocyte progenitor cells (OPCs) toward oligodendrocytes and requires high amounts of cholesterol. Astrocytes (ASTRs) modulate remyelination by supplying lipids to oligodendrocytes. Remarkably, remyelination is more efficient in grey matter (GM) than in white matter (WM), which may relate to regional differences in ASTR subtype. Here, we show that a feeding layer of gmASTRs was more supportive to in vitro myelination than a feeding layer of wmASTRs. While conditioned medium from both gmASTRs and wmASTRs accelerated gmOPC differentiation, wmOPC differentiation is enhanced by secreted factors from gmASTRs, but not wmASTRs. In vitro analyses revealed that gmASTRs secreted more cholesterol than wmASTRs. Cholesterol efflux from both ASTR types was reduced upon exposure to pro‐inflammatory cytokines, which was mediated via cholesterol transporter ABCA1, but not ABCG1, and correlated with a minor reduction of myelin membrane formation by oligodendrocytes. Surprisingly, a wmASTR knockdown of *Fdft1* encoding for squalene synthase (SQS), an enzyme essential for the first committed step in cholesterol biosynthesis, enhanced in vitro myelination. Reduced secretion of interleukin‐1β likely by enhanced isoprenylation, and increased unsaturated fatty acid synthesis, both pathways upstream of SQS, likely masked the effect of reduced levels of ASTR‐derived cholesterol. Hence, our findings indicate that gmASTRs export more cholesterol and are more supportive to myelination than wmASTRs, but specific inhibition of cholesterol biosynthesis in ASTRs is beneficial for wmASTR‐mediated modulation of myelination.

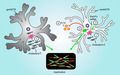

## Graphical abstract

Our findings revealed that regional differences in astrocytes (ASTRs) may contribute to the more efficient remyelination in grey matter (GM) than in white matter (WM). GmASTRs secrete more cholesterol (1) and are more supportive for in vitro myelination than wmASTRs. Surprisingly, impairing committed cholesterol biosynthesis in wmASTRs but not in gmASTRs increases in vitro myelination (2). The effect of decreased cholesterol levels is likely masked by enhanced synthesis of unsaturated fatty acid (3) and non‐sterol isoprenoids, the latter resulting in reduced secretion of ASTR‐derived cytokines, such as interleukin‐1β, that affect oligodendrocyte maturation (4). This may open new ASTR‐based therapeutic strategies aimed at promoting remyelination in multiple sclerosis lesions.

**Abbreviations**
ABCA1: ATP‐binding cassette transporter A1
ABCG1: ATP‐binding cassette transporter G1
ACM: astrocyte‐conditioned medium
ASTR: astrocyte ; BSA, bovine serum albumin
chol: cholesterol
CNS: central nervous system
ctrl: control
farnesyl‐PP: farnesyl‐pyrophosphate
FBS: fetal bovine serum
GFAP: glial fibrillary acidic protein
GM: grey matter
IFNγ: interferon gamma
IL1β: interleukin 1‐beta
kd: knockdown
LPS: lipopolysaccharide
MBP: myelin basic protein
MS: multiple sclerosis
NCM: non‐conditioned medium
NF: neurofilament‐H
OLG: oligodendrocyte
OPC: oligodendrocyte progenitor cell
PFA: paraformaldehyde
PLL: poly‐L‐Lysine
Poly(I:C): polyinosinic‐polycytidylic acid
RRID: research resource identifier
SCAP: SREBP‐cleavage‐activating protein
SQS: squalene synthase
SREBP: sterol regulatory element‐binding protein
T4: thyroxine
TLR: Toll‐like receptor
TNFα: tumor necrosis factor alpha
WM: white matter

## 1 INTRODUCTION

Multiple sclerosis (MS) is a demyelinating disease of the central nervous system (CNS) characterized by recurring inflammation, loss of oligodendrocytes (OLGs) and myelin, astrogliosis, remyelination failure, and neuronal loss (Compston & Coles, 2008; Reich, Lucchinetti, & Calabresi, 2018). Most therapies for MS do not directly aim to promote remyelination, but are rather immunomodulatory, reducing the number and severity of attacks (Plemel, Liu, & Yong, 2017). Remyelination is a regenerative process in which new myelin membranes are formed and which is not only essential to recover saltatory conduction, but also to prevent neurodegeneration (Franklin and ffrench‐Constant 2008; Fünfschilling et al., 2012; Irvine & Blakemore, 2008; Lee et al., 2012). MS lesions are present in both grey matter (GM) and white matter (WM) with GM remyelination being more pronounced than WM remyelination (Chang et al., 2012; Strijbis, Kooi, Valk, & van der, Geurts, 2017). Likewise, regional differences in remyelination efficiency are observed in experimental toxin‐induced demyelination models, where remyelination in the cortex, a GM area, is faster and more robust than remyelination in the corpus callosum, a WM area (Bai et al., 2016). Revealing the underlying mechanism of this apparent discrepancy between GM and WM remyelination may identify novel targets for therapy that promote remyelination.

In experimental models, remyelination proceeds via differentiation of oligodendrocyte progenitor cells (OPCs) to myelinating OLGs. Recent findings indicate that grey matter OPCs (gmOPCs) and white matter OPCs (wmOPCs) intrinsically differ (Lentferink, Jongsma, Werkman, & Baron, 2018; Viganò, Möbius, Götz, & Dimou, 2013). GmOPCs are less mature and more responsive to mitogens than wmOPCs (Lentferink et al., 2018), possibly rendering gmOPCs better equipped for remyelination (Moyon et al., 2015). In addition, wmOPCs are more susceptible to the detrimental effects of inflammatory mediators such as interferon‐γ (IFNγ, (Lentferink et al., 2018)). Astrocytes (ASTRs) play an important role in transient remodeling of the local signaling environment to facilitate remyelination upon CNS demyelination (Gudi, Gingele, Skripuletz, & Stangel, 2014; Jong, Wang, Oomkens, & Baron, 2020). Therefore, in addition to regional differences in OPCs, ASTRs from different brain regions may also contribute to regional differences in remyelination efficiency. Indeed, the cellular composition of GM and WM differ in the type of ASTRs that are present. More specifically, protoplasmic ASTRs are present in GM and fibrous ASTRs mainly reside in WM (Matyash & Kettenmann, 2010; Oberheim, Goldman, & Nedergaard, 2012; Schitine, Nogaroli, Costa, & Hedin‐Pereira, 2015). These ASTR subtypes are morphologically and functionally distinct, that is, protoplasmic ASTRs are morphological more complex and ensheath synapses, whereas fibrous ASTRs are specialized in providing structural support to myelinated axons and interact with the nodes of Ranvier (Oberheim et al., 2012). A dense astroglial scar is mainly formed in WM MS lesions and consists of persistent locally deposited extracellular matrix molecules, including proteoglycans and fibronectin that impair remyelination (reviewed in de Jong et al., 2020). While the role of ASTRs in remyelination in MS is still debated, a strong correlation exists between severe reactive glial scar formation and remyelination failure (Nair, Frederick, & Miller, 2008; Nash, Ioannidou, & Barnett, 2011). Hence, ASTRs from GM and WM may distinctly contribute to remyelination efficiency in MS lesions.

When OLGs are unable to produce cholesterol during CNS development myelin formation is reduced, while there is no difference in the ratio of cholesterol to other lipids incorporated in myelin (Saher et al., 2005). This indicates that cholesterol is rate‐limiting for myelin membrane growth and an indispensable lipid component of myelin membranes. ASTRs are the main suppliers of cholesterol in the CNS (Camargo et al., 2012, 2017; Dietschy & Turley, 2004; Nieweg, Schaller, & Pfrieger, 2009; Pfrieger, 2003) and likely supply cholesterol to OLGs via lipoprotein particles (Saher et al., 2005). Although OPCs synthesize more cholesterol upon toxin‐induced demyelination (Voskuhl et al., 2019), remyelination may also benefit from ASTR‐derived cholesterol (Camargo et al., 2012). Indeed, both cholesterol supplementation and enhancing cholesterol efflux in toxin‐induced demyelination models, accelerates OPC differentiation and remyelination in the corpus callosum, emphasizing a role of horizontal cholesterol transfer for remyelination (Berghoff et al., 2017; Cantuti‐Castelvetri et al., 2018). In addition, when remyelination is impaired, such as in experimental autoimmune encephalomyelitis and in MS, genes encoding for enzymes involved in cholesterol production, including *Hmcgs1*, *Fdps,* and *Fdft1,* are decreased in ASTRs (Itoh et al., 2018). Here, we aimed to address whether gmASTRs and wmASTRs differ in cholesterol production and/or efflux and thereby distinctly modulate myelination. Our findings revealed that although the availability of ASTR‐derived cholesterol may play a role in OPC maturation, downregulation of committed cholesterol biosynthesis in wmASTRs but not gmASTRs, unexpectedly led to increased myelination in vitro.

## 2 MATERIALS AND METHODS

### 2.1 Primary cell cultures

Animal protocols were approved by Animal Ethics Committee of the University of Groningen (the Netherlands, 16504‐01‐02). All animals were used for cell culture experiments and carried out in accordance with the European Directive (2010/63/EU) on the protection of animals used for scientific purposes. Timed‐mated female Wister rats were purchased from Harlan (RCCHan:WIST, RRID:RGD_5508396) or Charles River (CRL:WI(WU), RRID:RGD_2312472) and kept under standardized temperature and humidity and a 12 hr light–dark cycle with water and food ad libitum in standard cages (one pregnant female rat or one female rat with pups per cage). A total of approximately 30 pregnant female rats were used. The dissected brains or spinal cords from 4 to 12 newborn rats (both genders) of one litter were pooled and considered as one independent cell culture preparation. There were no exclusion criteria predetermined, the study was exploratory and not pre‐registered. Cell treatments were not performed in a blinded manner, and the number of experiments was not predetermined.

#### 2.1.1 Oligodendrocytes

Newborn rats (postnatal day 1–3) were decapitated without anesthesia. Primary OPCs were isolated from the cortex (GM, referred to as gmOPCs) and non‐cortical parts (referred to as wmOPCs, WM tracts including corpus callosum, mixed GM and WM tracts, including hippocampus and thalamus, and deep GM parts, including basal ganglia), of forebrains using a shake‐off procedure as described (Baron, Shattil, & ffrench‐Constant C., 2002; Bsibsi, Nomden, Noort, & van, Baron W., 2012; Lentferink et al., 2018). Upon 12–14 days in mixed glial cultures, contaminating microglia were removed from the confluent ASTR layer by a pre‐shake at 150 rpm on an orbital shaker (Innova 4,000, New Brunswick) for one hour at 37°C. To obtain the more firmly attached OPCs, flasks were shaken at 240 rpm overnight at 37°C. The detached OPCs were further purified by differential adhesion on non‐tissue culture dishes for 15–20 min. The enriched OPC fraction contained 95%–97% OPCs (Olig2‐positive, Millipore, RRID:AB_570666), less than 1% microglia (IB4‐positive, Invitrogen, I21412), 1%–3% ASTRs (glial fibrillary acidic protein (GFAP)‐positive, DAKO, RRID:AB_10013382) and less than 1% neurons (TuJ1‐positive, kind gift of Dr A. Frankfurter, University of Virginia). OPCs were cultured on 13‐mm poly‐L‐lysine (5 µl/ml; Sigma, P2636)‐coated glass coverslips in 24‐well plates at a density of 35,000 cells/well (gmOPCs) or 40,000 cells/well (wmOPCs) in defined Sato medium (Lentferink et al., 2018). OPCs were synchronized to early OPCs by 10 ng/ml platelet‐derived growth factor‐AA (PDGF‐AA; Peprotech, 100–13) and 10 ng/ml human fibroblast growth factor‐2 (FGF‐2; Peprotech, 100–18). After two days, growth factors were removed and OPCs were allowed to differentiate in Sato medium supplemented with 0.5% fetal bovine serum (FBS, Capricorn, FBS‐12A) for three or six days in the absence or presence of astrocyte‐conditioned medium (ACM, 1:1).

#### 2.1.2 Astrocytes

After obtaining OPCs, remaining ASTRs of the mixed glial cultures, were shaken at 240 rpm overnight at 37°C, passaged once by trypsinization, transferred to 162 cm^2^ flasks (Corning, 3,150) and cultured in ASTR medium [100 U/ml penicillin and 100 μg/ml streptomycin (Gibco, 15,140,122, 4 mM L‐glutamine (Invitrogen, 25030‐123), 10% heat‐inactivated FBS (Bodinco, 4005‐BDC‐0814) in DMEM (Gibco, 49,165,039)]. This yielded a highly pure ASTR population for both gmASTRs and wmASTRs (>97% GFAP‐positive). For immunocytochemistry, ASTRs were plated on poly‐L‐lysine‐coated glass coverslips at a density of 5.0 × 10^4^ cell/well in a 24‐well plate. For cholesterol assays and qPCR assays, ASTRs were cultured at a density of 1.0 × 10^6^ cells/well in six‐well plates in ASTR medium. For cholesterol assays, cells were washed after 24 hr and serum‐free ASTR medium was added. In case of lentiviral transduced ASTRs, defined SATO medium was added. ASTRs were either left untreated or treated for 24 hr with either Toll‐like‐receptor (TLR) 3 agonist polyinosinic‐polycytidylic acid (Poly(I:C), 50 μg/mL, Sigma, P1530), TLR4 agonist lipopolysaccharide (LPS, 200 ng/ml, Sigma, L4391), or a combination of the following pro‐inflammatory cytokines: IFNγ (500 IU/mL, Peprotech, 400–20), interleukin‐1β (IL1β, 1 ng/ml, Peprotech, 400‐01), and tumor necrosis factor‐α (TNFα, 10 ng/ml, Peprotech, 400‐14). To inhibit cholesterol transporters, either ATP‐binding cassette transporter (ABC) A1 inhibitor glibenclamide (0.1 mM, Sigma, G0639) or ABCG1 inhibitor thyroxine (T4, 50 µM, Sigma, T1775) were added one hour before addition of cytokines. For immunoblot analysis, 1.0 × 10^6^ cells were plated on 10 cm dishes (Corning, 430,167). The cells were either left untreated or treated with a mixture of pro‐inflammatory cytokines (IFNγ, IL1β, and TNFα) for 24 hr at 37°C. For collection of ACM, cells were plated in 6‐well plates at 1.0 × 10^6^ cells/well in ASTR medium. ASTRs were either left untreated or treated with a mixture of pro‐inflammatory cytokines IFNγ, IL1β, and TNFα, or cultured in the presence of 10 μg/ml bodipy‐cholesterol (Avanti, 8110255, Hölttä‐Vuori et al., 2008). After 24 hr, cells were washed and cultured for another 24 hr in Sato medium. ACM was collected, filtered using a 0.45 µm filter (GE Healthcare, 10462100) and stored at −20˚C.

#### 2.1.3 Spinal cord cultures

Myelinating spinal cords were generated from 15‐days old rat embryos as described with minor modifications (Sorensen, Moffat, Thomson, & Barnett, 2008). Pregnant female rats were sacrificed with overdoses of euthasol via IC injection when under anesthesia (5% isoflurane), after which the embryos were removed and decapitated. After removal of the meninges from the isolated spinal cords, tissue was mechanically dissociated in Leibowitz L‐15 medium (Sigma, L4386) followed by enzymatic digestion with a mixture of trypsin (2.5%, Sigma, T8253) and DH liberase (2.5 mg/ml, Roche, 05401054001) for 20 min at 37°C. The enzymatic reaction was stopped by addition of Soybean trypsin inhibitor solution [0.52 mg/ml soybean trypsin inhibitor (Sigma, T6522), 0.04 mg/ml bovine pancreas DNase (Roche, 10104159001) and 3 mg/ml fatty acid‐depleted bovine serum albumin (BSA, Sigma, A4919) made up in Leibovitz's L15 medium]. The cells were centrifuged for 7 min at 180 g, followed by resuspension in plating medium consisting of 50% DMEM (1,500 mg/L glucose, Gibco, 31885023), 25% horse serum (Invitrogen, 26050088), 25% HBSS with calcium and magnesium (Invitrogen, 14025‐100), and 2 mM L‐glutamine. Cells were plated on 13‐mm glass coverslips (VWR, 631‐0150) in 24‐well plates at a density of 200,000 cells/well or in an 8‐well permanox chamber slide (Nunc, 177445) at 160,000 cells/well in respectively 500 μl or 200 μl plating medium. The coverslips and chamber slides contained a confluent feeding layer of gmASTRs or wmASTRs (80,000 cells/13‐mm coverslip; 64,000 cells/chamberslide well). After 2 hr, the volume of the medium was doubled with growth medium [DMEM 4,500 mg/L glucose, (Gibco, 49,165,039) supplemented with 5 mg/ml holotransferin (Sigma, T0665), 20 mM putrescine (Sigma, P5780), 4 µM progesterone (Sigma, P‐0130), 6 µM selenium (Sigma, S5261), 10 ng/ml biotin (Sigma, B4639), 50 nM hydrocortisone (Sigma, H0135), and 10 μg/mL insulin (Sigma, I1882)], and when cultured on permanox chamber slides supplemented with 27.5 µM 2‐mercaptoethanol (Gibco, 21985023). Every two to three days, half of the medium was replaced by new growth medium. Insulin was omitted from growth medium from 12 days in cultures onwards. Cultures were analyzed after 28 days in culture.

### 2.2 Lentivirus production and transduction

For production of lentiviral particles, the constructs (pLKO.1‐puro Sigma mission shRNA, construct SHCLNG‐NM_010191, sequence TRCN0000099191, for rat *Fdft1* shRNA, or SHC002 for control scrambled shRNA; 5 µg), packaging, and envelope plasmids (500 ng pMD2‐VSVG; 5 µg pCMV‐R8.91) were transfected into the HEK293T packaging cell line using Fugene (Promega, E2311) according to manufacturer's instructions. After 16 hr, medium was changed to mixed glial culture medium [100 U/ml penicillin, 100 μg/ml streptomycin, 4 mM L‐glutamine, 10% (v/v) FBS in DMEM]. Viral particles were harvested in mixed glial culture medium for 48 hr and filtered through polyvinylidene fluoride (PVDF) membrane‐based 0.45‐μm filter (GE Healthcare, 514–0226) and either used immediately or stored at −80°C. Lentiviral particles were added overnight to ASTRs (1:3 with ASTR medium) in the presence of hexadimethrine bromide (Polybrene, 8 μg/mL, Sigma, TR1003). Cells were washed and either used for spinal cord cultures or left for five days for qPCR analysis, cholesterol assays and production of ACM.

### 2.3 Cholesterol assays

#### 2.3.1 Lipid extraction

Lipids from cells and medium were extracted using the Bligh and Dyer method (Bligh & Dyer, 1959; Klappe, Hummel, & Kok, 2013). Briefly, a methanol‐chloroform‐mixture (1:2; chloroform, Merck, 1.02445; methanol, Biosolve, 136806) was added and the samples were centrifuged at 820 g for 5 min. The lower phase was collected and the upper phase was further processed by adding chloroform (1:1). After centrifugation at 820 g for 5 min, the lower phase was added to previous collected lower phase and dried with a vacuum centrifuge and/or heating to 60°C. To determine cholesterol and phosphate levels, the dried lipid extracts of the cell samples were dissolved in 0.5 ml chloroform mixture (1:1), split and dried with the vacuum centrifuge.

#### 2.3.2 Cholesterol levels

Free cholesterol levels in cells (intracellular) and medium (extracellular) were quantified using a fluorescence‐based enzymatic method (Gamble, Vaughan, Kruth, & Avigan, 1978; Klappe et al., 2013). The dried lipid extracts were dissolved in ethanol and a mixture of 0.63 mg/ml parahydroxy‐phenylacetic acid (Sigma, H50004), 0.2 M phosphate buffer (pH 7.4), 20 mM sodium cholate (Sigma, C9282), 0.5% Triton‐X‐100, water, cholesterol oxidase (0.15 U/mL, Sigma, 9028‐76‐6) and peroxidase (0.95 U/mL, Sigma, P6140) was added and incubated for 20 min in the dark. Of note, cholesterol oxidase is sensitive to cholesterol, and oxidizes other sterols with a much lower rate (MacLachlan, Wotherspoon, Ansell, & Brooks, 2000). Standard concentrations of 0–29 nmol cholesterol were also included. Fluorescence was measured with an excitation of 325 nm and an emission of 415 nm (PerkinElmer instruments LS 55). The cholesterol efflux was calculated using the formula: efflux = extracellular cholesterol levels/ (intracellular + extracellular cholesterol levels).

#### 2.3.3 Phosphate determination

As an internal control, the amount of phosphate in each sample was determined serving as an indication of the lipid amount in the sample (Smith et al., 1985). To this end, a standard curve was prepared using 0–320 nmol phosphate. Then, 0.2 ml of 70% perchloric acid (Sigma, 244252) was added and the samples were heated to 180°C for 30 min. After cooling down, 2 ml of molybdate reagent (ammonium heptamolybdate tetrahydrate (Sigma, 12054–85–2), concentrated sulfuric acid (Sigma, 7664‐93‐9), water) and 0.25 ml of freshly made 10% ascorbic acid (Wako Pure Chemical, 323‐44822) were added and the samples were heated to 95°C for ten minutes. To stop the reaction, the samples were placed in ice and absorbance was measured at 812 nm (μQuant, Bio‐Tek). For intracellular cholesterol levels, the amount of cholesterol was normalized to phosphate, that is, the ratio of cholesterol to phosphate was calculated (nmol/nmol).

### 2.4 Western blot analysis

For immunoblots of ABCA1 and ABCG1, cells were washed with phosphate‐buffered saline (PBS) and scraped in lysis buffer [1% Triton X‐100, 50 mM Tris‐HCl, 150 mM NaCl, 5 mM EDTA, and protease inhibitor cocktail (Roche, 11836153001), pH 7.4]. Protein concentration was determined by a detergent‐compatible protein determination assay (Bio‐Rad, 500–0116) according to manufacturer's instructions using BSA as a standard. Equal amounts of proteins (50 μg) were loaded onto 7.5% SDS‐polyacrylamide gels. For detection of IL1β in medium, equal volumes (80 µl) were loaded onto 15% SDS‐polyacrylamide gels. After gel electrophoresis, proteins were transferred onto PVDF membranes (Millipore, IPFL00010) via wet transfer. After blocking the membrane for one hour with Odyssey blocking buffer (1:1 in PBS, Li‐Cor Biosciences, 927‐40003), membranes were incubated with primary antibodies against ABCA1 (monoclonal mouse anti‐ABCA1, 1:500, Novus Biologicals, RRID:AB_10002789), ABCG1 (polyclonal rabbit anti‐ABCG1, 1 μg/ml, Novus Biologicals, RRID:AB_10125717), actin (monoclonal mouse anti‐β‐actin; 1:2000, Sigma, RRID:AB_476744), or IL1β (monoclonal hamster anti‐IL1β, 1:200, Santa Cruz, RRID:AB_627791) overnight at 4°C. After washing with PBS containing 0.1% Tween‐20, IRDye‐conjugated secondary antibodies (Li‐Cor Biosciences, Lincoln, RRID:AB_10956588, RRID:AB_10956166, RRID:AB_621843, and RRID:AB_621842; 1:3,000) were incubated for one hour. For IL1β, an additional incubation step with a rabbit‐anti‐hamster linker antibody (Jackson, RRID:AB_2339572) was conducted, before secondary antibodies were applied. The bands were visualized with the Odyssey Imaging System (Li‐Cor). For cell lysates, the expression of each protein was calculated relative to the amount of β‐actin with densitometry using FIJI (ImageJ).

### 2.5 Immunocytochemistry

#### 2.5.1 Primary cell cultures

The cells were fixed with 4% paraformaldehyde (VWR, 1.04005) for 15 min at 20 ℃, and permeabilized with ice‐cold methanol for 10 min. Non‐specific antibody binding was blocked with 4% BSA for 30 min after which cells were incubated with primary antibodies anti‐myelin basic protein (MBP, 1:250, Serotec, RRID:AB_325004) or anti‐GFAP (1:500) at 20 ℃. The cells were rinsed three times with PBS before the appropriate Alexa Fluor©‐conjugated secondary antibodies (1:500, Thermo Fisher Scientific, RRID:AB_2534093, RRID:AB_2534125) were added together with DAPI (1 μg/ml, nuclear stain, Sigma, D9542) for 30 min at 20 ℃. After three washes with PBS, coverslips were mounted using mounting medium (Dako, S302580–02). Cells were analyzed using a conventional immunofluorescence microscope (Leica DMI 6,000 B) equipped with Leica Application Suite Advanced Fluorescence software. In each independent experiment, approximately 150–250 cells per coverslip were scored for the percentage of MBP‐positive cells of DAPI‐stained cells (differentiation), or for the percentage of MBP‐positive cells that form myelin membranes (myelin membrane formation).

#### 2.5.2 Spinal cord cultures

Spinal cord cultures were fixed with 4% paraformaldehyde for 30 min, and blocked and permeabilized with 0.1% Triton X‐100 in 4% BSA in PBS for 45 min. After three washes with PBS, cells were incubated for 90 min with anti‐MBP (1:250) and anti‐neurofilament‐H (NF, polyclonal chicken anti‐neurofilament, 1:5,000, EnCor Biotechnology Inc.,RRID:AB_2149761) antibodies at 20 ℃. Cells were rinsed three times with PBS before the appropriate FITC‐conjugated secondary antibodies (1:50, Jackson Immunolaboratories, RRID:AB_2338189) or Alexa Fluor©‐conjugated secondary antibodies (1:500, RRID:AB_2534074, RRID:AB_2534098) were added together with DAPI for 45 min at 20 ℃. Coverslips and slides were mounted with mounting medium (Dako). Samples were imaged using either a conventional immunofluorescence microscope (Leica DMI 6,000 B) or a confocal microscope (TCS SP2 or SP8 AOBS Microscope, Leica Microsystems) using Leica Software. The percentage of myelinated axons was either calculated in ImageJ as an area in pixels in each image occupied by both myelin and axons divided by the axonal density (Qin et al., 2017; Stancic et al., 2012) or in MATLAB using software programmed to recognize only linear structures, thus only including myelin and axons and excluding OLG cell bodies (Qin et al., 2017). In each experiment, five images per coverslip and one to two coverslips per condition were analyzed.

### 2.6 qPCR analysis

The cells were scraped in RNA protect (Qiagen, 76526) and RNA was isolated using an RNA‐isolation kit (Isolate II RNA Micro Kit; Bioline, BIO‐52075) according to manufacturer's instructions. RNA (1 μg) was reverse transcribed in the presence of oligo(dT)12–18 (Invitrogen, 18418012) and dNTPs (Invitrogen, 10297018) with M‐MLV reverse transcriptase (Invitrogen, 28025013). mRNA levels of *Fdft1*, *Fasn*, *Srebf1c*, and *Srebf2* were measured by real‐time quantitative reverse transcriptase PCR (qPCR) using Absolute qPCR SYBR Green Master Mix (BioRad, 172‐5124) in a Step‐One Plus Real‐Time PCR machine. Each measurement was performed in triplicate and amplification data was processed using the LinRegPCR method (Ramakers, Ruijter, Lekanne, & H., Moorman A. F. M., 2003; Ruijter et al., 2009). Primer sequences are shown in Table 1. Expression was normalized to housekeeping genes *Eef1a1* or *Hprt1*.

**Table 1:** Primer sequences used for qPCR

gene	Forward primer	Reverse primer	Product size (bp)
*Eef1a1*	5’‐GATGGCCCCAAATTCTTGAAG−3’	5’‐GGACCATGTCAACAATTGCAG−3’	52
*Fdft1*	5’‐TCTACAACCTGCTGCGATTC−3’	5’‐GCGACTGGTCTGATCAAGATAC−3’	119
*Fasn*	5’‐GGCAATACCCGTTCCCTGAA−3’	5’‐GGCAATACCCGTTCCCTGAA−3’	92
*Hprt1*	5’‐GACTTGCTCGAGATGTCA−3’	5’‐TGTAATCCAGCAGGTCAG−3’	102
*Srebf1c*	5’‐GGAGCCATGGATTGCACATTT−3’	5’‐CCAGCATAGGGGGCATCAAA−3’	92
*Srebf2*	5'‐GGGCTGTCGGGTGTCATGG−3'	5'‐GGCAATACCCGTTCCCTGAA−3'	105

### 2.7 Statistical analysis

Data are expressed as mean ± standard error of the mean (*SEM*) of at least three independent cell culture preparation experiments. A Shapiro–Wilk normality test was first applied to test the normal distribution of the data. When normality failed, a Kruskal–Wallis test was used to test for statistical significance. When normality passed and when comparing absolute values between groups (i.e., gmASTRs vs. wmASTRs) statistical significance was assessed using a paired two‐sided *t*‐test. When normality passed and when relative groups were compared to control [i.e., non‐conditioned medium (NCM), control gmASTRs, control wmASTRs, control gmACM, or control wmACM], statistical analysis was performed with a one‐sample *t*‐test, with the indicated control set to 1 in each independent cell culture experiment. A paired student's *t*‐test was used to test for differences between effects of wmACM and gmACM on OPC differentiation (^#^) with NCM was set to 1 in each independent cell culture experiment. A one‐way ANOVA with a Šidák multiple comparisons post‐test was performed to test for differences between multiple different treatments. Statistics were performed using GraphPad Prism 6.0. In all cases, *p*‐values of < .05, <0.01, and < .001 were considered significant and indicated with *, ** and *** or ^#^, ^##^, and ^###^, respectively.

## 3 RESULTS

### 3.1 Cultured grey matter astrocytes are more supportive to myelination than cultured white matter astrocytes

**FIGURE 1:**
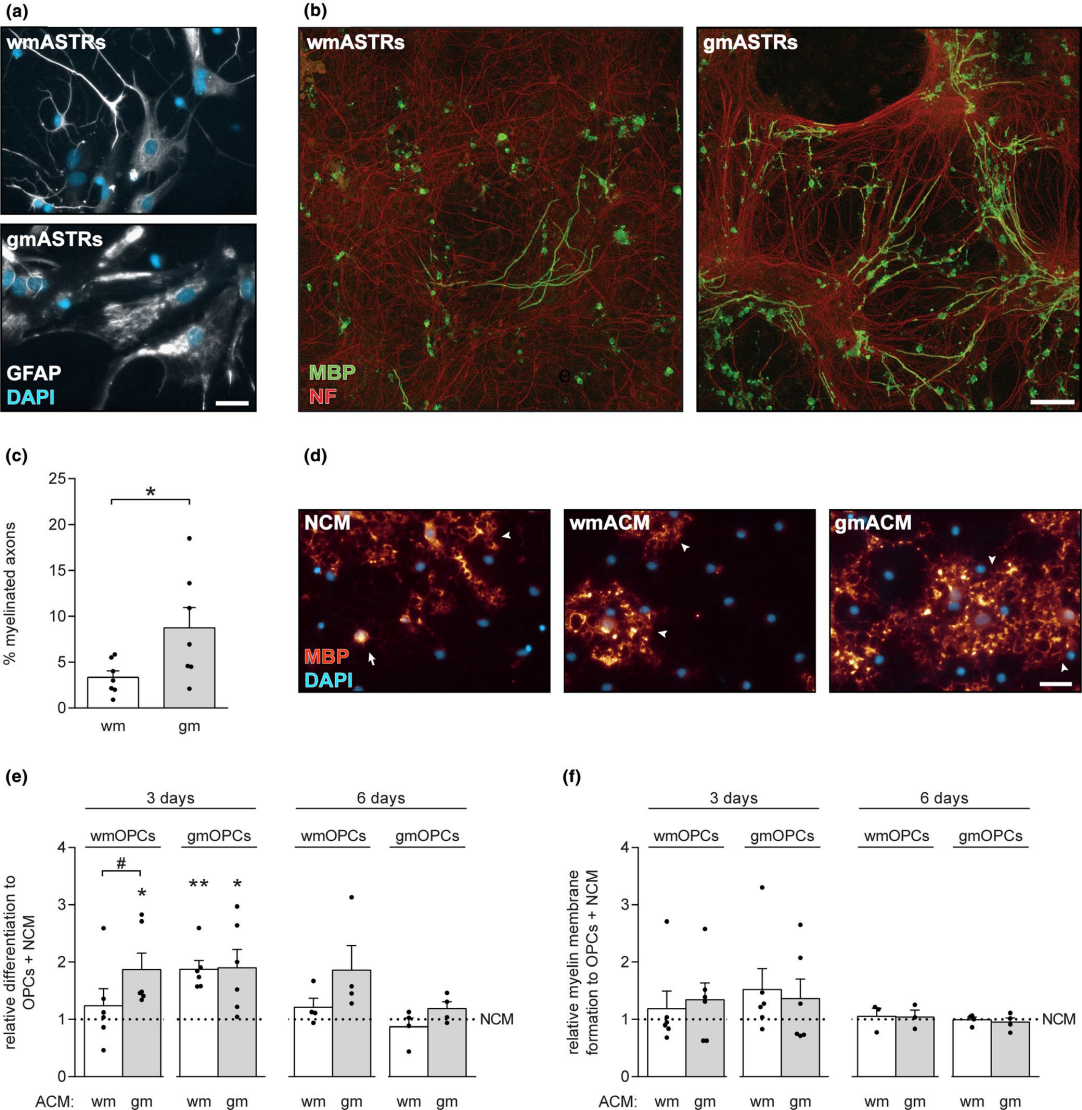
Grey matter astrocytes are more supportive for in vitro myelination than white matter astrocytes. (a) GFAP immunocytochemistry of primary neonatal grey matter (gm) and white matter (wm) astrocytes (ASTRs). (b and c) In vitro myelinating cultures that depend on a feeding layer of ASTRs are obtained from 15‐day‐old rat embryo spinal cord cells and stained for the myelin marker myelin basic protein (MBP, green) and the axonal marker neurofilament‐H (NF, red). Representative images are shown in (b) and quantification of the percentage of myelinated axons in (c) (*n* = 6 independent cell culture preparations). Note that a feeding layer of gmASTRs is more supportive for in vitro myelination than a feeding layer of wmASTRs (*p* = .018). (d‐f) Neonatal wmOPCs (d–f) and gmOPCs (e and f) were differentiated for three or six days in the presence of non‐conditioned medium (NCM) or ASTR‐conditioned medium (ACM) from gmASTRs or wmASTRs. MBP immunocytochemistry is performed to assess differentiation (% MBP‐positive cells of DAPI‐stained cells) and myelin membrane formation (% myelin membranes formed by MBP‐positive cells). Representative images of MBP‐positive wmOLGs (red) in the presence of NCM, wmACM or gmACM three days after initiating differentiation are shown in (d) (arrow indicates MBP‐positive cells; arrowheads point to myelin membranes). Quantification of OPC differentiation in the presence of NCM, wmACM or gmACM is shown in (e) and quantification of myelin membrane formation in (f) (*n* = 4–6 independent cell culture preparations). Note that upon exposure to gmACM but not wmACM, wmOPC differentiation is significantly increased three days after initiating differentiation (*p* = .028) compared to exposure to NCM, while gmOPC differentiation is increased upon exposure to both wmACM and gmACM (wmACM *p* = .002, gmACM *p* = .037). Bars represent absolute values (c) or relative means compared to NCM (e,f), which is set to 1 in each independent experiment. Error bars represent standard error of the mean (*SEM*). Statistical analyses are performed using column statistics with a one‐sample *t*‐test (*p < .05) to test for differences between ACM treatments and NCM‐treated control, while a paired *t*‐test (*p < .05) is used to test for differences between effects of gmACM and wmACM (not significant). For wmOPC differentiation absolute values of NCM are 15.7 ± 3.5% after three days and 36.5 ± 9.1% after six days and for myelin membrane formation 55.5 ± 5.6% after three days and 63.5 ± 16.9% after six days. For gmOPC differentiation absolute values of NCM are 9.0 ± 2.7% after three days and 47.5 ± 6.1% after six days and for myelin membrane formation 47.8 ± 8.9% after three days and 84.2 ± 2.1% after six days. Scale bars are 25 µm (a and d) and 50 µm (b)

To examine whether gmASTRs and wmASTRs distinctly modulate myelination efficiency, an in vitro myelinating system of embryonic spinal cord cultures that relies on an ASTR feeding layer was employed (Qin et al., 2017; Sorensen et al., 2008). To this end, ASTRs from the cerebral cortex (GM, referred to as gmASTRs) and non‐cortical parts (mainly WM, referred to as wmASTRs) of postnatal day 1–3 rat forebrains were isolated and cultured for at least 21 days in a mixed glial culture. The obtained gmASTRs and wmASTRs were positive for the ASTR marker GFAP (Figure 1a). Consistent with previous observations of cultured ASTRs isolated from different brain regions at postnatal day 5 (Goursaud, Kozlova, Maloteaux, & Hermans, 2009), gmASTRs were more protoplasmic, while wmASTRs adopted a more fibrous stellate morphology (Figure 1a). Therefore, regional differences in morphology are likely acquired even before ASTRs are mature, that is, ASTRs are considered mature at postnatal day 14–21 (Bushong, Martone, & Ellisman, 2004; Cahoy et al., 2008), likely as ASTRs do not postnatally redistribute (Bayraktar, Fuentealba, Alvarez‐Buylla, & Rowitch, 2014; Taft, Vertes, & Perry, 2005; Tsai et al., 2012). Assessment of the percentage of myelinated axons, as determined by a co‐labeling of the myelin marker MBP and the axonal marker NF, revealed that the percentage of myelinated axons was higher on a feeding layer of gmASTRs than on a feeding layer of wmASTRs (Figure 1b and c, *p* = .018). In monoculture, OPCs readily differentiate into MBP‐positive OLGs that elaborate myelin membranes. To examine whether the distinct modulation of protoplasmic gmASTRs and fibrous wmASTRs on in vitro myelination relates to a difference in gmASTR‐ and wmASTR‐derived secreted factors, the effect of astrocyte‐conditioned medium (ACM) on OPC differentiation and myelin membrane formation was examined in monocultures. As gmOPCs and wmOPCs distinctly respond to injury signals (Lentferink et al., 2018), and as both are present in our in vitro myelinating cultures, the effect of ACM from gmASTRs (gmACM) or wmASTRs (wmACM) on both gmOPC and wmOPC maturation was taken into account. Three days after initiating wmOPC differentiation, exposure to gmACM, but not wmACM, increased the percentage of MBP‐positive cells (Figure 1e, *p* = .028) ‐a read‐out for OPC differentiation‐ compared to non‐conditioned medium (NCM). Moreover, six days after initiating wmOPC differentiation, the percentage of MBP‐positive cells was reproducibly, but not significantly higher in the presence of gmACM than in the presence of wmACM (Figure 1e), indicating that gmACM, but not wmACM, enhanced wmOPC differentiation. In contrast, three days after initiating gmOPC differentiation the percentage of MBP‐positive cells was significantly enhanced upon exposure to both gmACM and wmACM compared to NCM (Figure 1e, wmACM *p* = .002; gmACM *p* = .037). Remarkably, six days after initiating gmOPC differentiation, the percentage of MBP‐positive cells was similar at all conditions, indicating that both wmACM and gmACM accelerated gmOPC differentiation. No significant difference in myelin membrane formation was observed upon gmACM and wmACM treatment of either type of OPC at three days or six days after initiating differentiation (Figure 1f). Hence, these findings demonstrated that gmASTRs were more supportive to in vitro myelination than wmASTRs, likely by secreting (more) factors that enhanced wmOPC differentiation. As ASTRs are the most important lipid suppliers in the adult CNS (Camargo et al., 2017) and exogenously supplied cholesterol accelerates OPC differentiation (Berghoff et al., 2017), we next examined whether gmASTRs and wmASTRs differ in their capacity to supply cholesterol to differentiating OLGs.

### 3.2 Grey matter astrocytes secrete more cholesterol than white matter astrocytes

**FIGURE 2:**
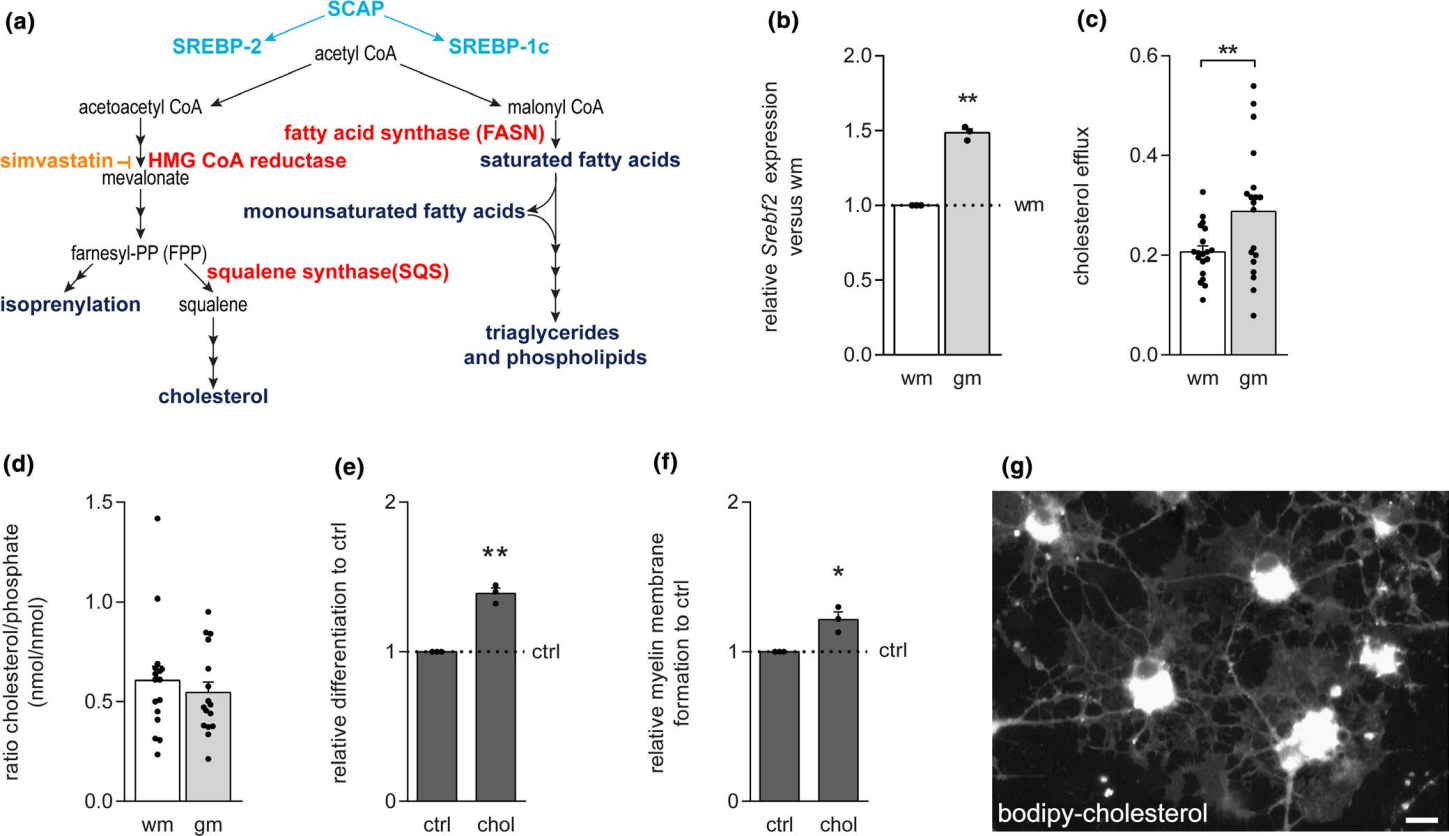
Grey matter astrocytes secrete more cholesterol than white matter astrocytes. (a) Schematic overview of several intermediates in the cholesterol and unsaturated fatty acid synthesis pathway. SREBP‐2 and SREBP‐1c are transcription factors, and SQS and FASN are enzymes involved in committed cholesterol and unsaturated fatty acid synthesis, respectively. (b) qPCR analysis of *Srebf2* in primary neonatal grey matter (gm) and white matter (wm) astrocytes (ASTRs; *n* = 3 independent cell culture preparations). (c and d) Cholesterol assays on gmASTRs and wmASTRs. Cholesterol efflux is shown in (c) and intracellular cholesterol levels, shown as intracellular cholesterol/phosphate ratios, in (d) (*n* = 16–19 independent cell culture preparations). (e and f) Neonatal wmOPCs were differentiated for three days in the absence (ctrl) or presence of 10 μg/ml cholesterol (chol). Myelin basic protein (MBP) immunocytochemistry is performed to assess differentiation (e, % MBP‐positive cells of DAPI‐stained cells) and myelin membrane formation (f, % myelin membranes formed by MBP‐positive cells; *n* = 3 independent cell culture preparations). Note that exposure to cholesterol enhances wmOPC differentiation (*p* = .008) and myelin membrane formation (*p* = .048). (g) wmOPCs were exposed to ACM obtained from ASTRs that were pre‐incubated with 10 μg/mL bodipy‐cholesterol and analyzed three days after initiating differentiation. Note that wmOLGs take up bodipy‐cholesterol secreted from ASTRs. Bars represent absolute values (c and d) or relative means compared to wmASTRs (b, wm) or untreated wmOLGs (e and f, ctrl), which are set to 1 in each independent experiment. Error bars represent standard error of the mean (*SEM*). Statistical analyses are performed using column statistics with a one‐sample *t*‐test (*p < .05, **p < .01) to test for differences with wmASTRs (b) or ctrl wmOLGs (e and f), while a paired student *t*‐test (**p < .01) is used to test for differences between cholesterol efflux from wmASTRs and gmASTRs. Absolute values of ctrl OLGs for OPC differentiation are 20.5 ± 4. 1% and for myelin membrane formation 46.7 ± 6.4%. Scale bar is 10 µm

Sterol regulatory element‐binding proteins (SREBPs) are a family of membrane‐bound transcription factors that modulate the transcription of genes of enzymes that are required for the synthesis of cholesterol and unsaturated fatty acids (Shimano & Sato, 2017; Shimomura, Shimano, Korn, Bashmakov, & Horton, 1998). SREBP‐2 drives the transcription of genes of enzymes involved in cholesterol biosynthesis (Figure 2a). qPCR analysis revealed that mRNA levels of SREBP‐2 (*Srebf2)* were higher in cultured gmASTRs than in cultured wmASTRs (Figure 2b, *p* = .003). In addition, cholesterol efflux from gmASTRs was higher than cholesterol efflux from wmASTRs (Figure 2c, *p* = .006), while intracellular levels of cholesterol in gmASTRs and wmASTRs were comparable (Figure 2d). Other studies have demonstrated that exogenously supplied cholesterol facilitates mouse gmOPC differentiation in vitro (Berghoff et al., 2017). To confirm this observation for rat wmOPCs, rat wmOPCs were differentiated in the presence of cholesterol. Three days after initiating differentiation, both wmOPC differentiation (Figure 2e, *p* = .008) and myelin membrane formation (Figure 2f, *p* = .048) were enhanced upon cholesterol treatment. Labeling of ASTRs with the fluorescent cholesterol derivative bodipy‐cholesterol (Hölttä‐Vuori et al., 2008), followed by exposure of differentiating wmOPCs to ACM, showed that ASTR‐derived bodipy‐cholesterol was taken up by wmOLGs (Figure 2g). Thus, cholesterol efflux was higher from gmASTRs, which strongly suggests that the increased levels of secreted cholesterol by gmASTRs may add to enhanced wmOPC differentiation in the presence of gmACM, but not wmACM (Figure 1e). As inflammatory mediators interfere with cholesterol efflux (Yin, Liao, & Tang, 2010) and to further explore the role of ASTR‐derived cholesterol in myelination efficiency, the effect of MS‐relevant inflammatory mediators on cholesterol efflux from ASTRs was examined next.

### 3.3 A mixture of pro‐inflammatory cytokines reduces cholesterol efflux from grey matter and white matter astrocytes

**FIGURE 3:**
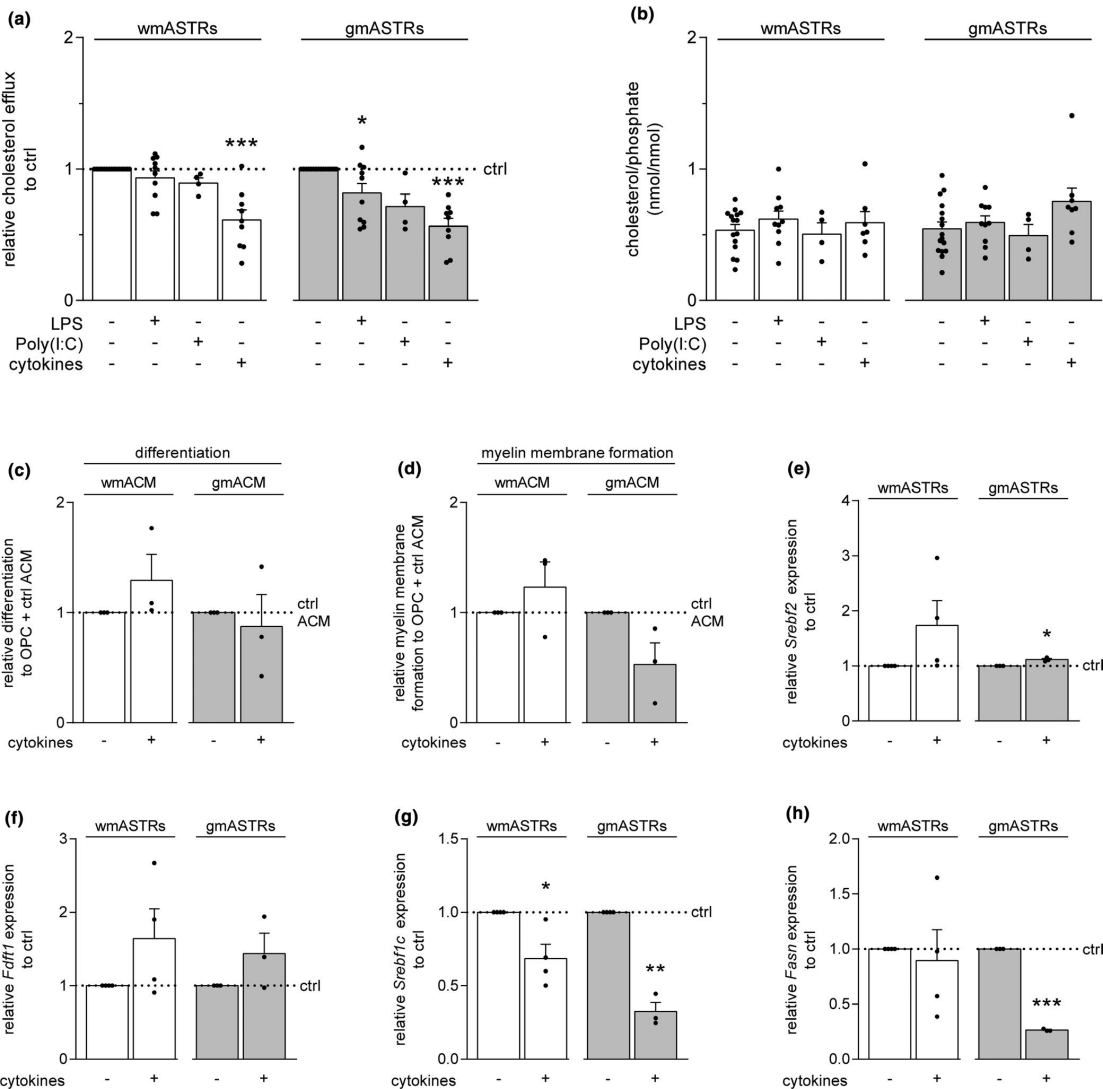
A mixture of pro‐inflammatory cytokines reduces cholesterol efflux from grey and white matter astrocytes. Primary neonatal grey matter (gm) and white matter (wm) astrocytes (ASTRs) were either untreated (ctrl) or treated for 24 hr with TLR4 agonist LPS (200 ng/ml), TLR3 agonist Poly(I:C) (50 μg/ml), or a mixture of pro‐inflammatory cytokines consisting of IL1β (1 ng/ml), IFNγ (500 U/ml) and TNFα (10 ng/ml). (a) Cholesterol assays on gmASTRs and wmASTRs. Relative cholesterol efflux is shown in (a) and intracellular cholesterol levels, shown as intracellular cholesterol/phosphate ratios of gmASTRs and wmASTRs, in (b) (*n* = 4–15 independent cell culture preparations). Note that treatment of gmASTRs with LPS (*p* = .033) as well as treatment of both types of ASTRs with the pro‐inflammatory cytokine mix (wmASTRs *p* = .001; gmASTRs *p *≤ 0.001) results in a decrease of cholesterol efflux, while intracellular cholesterol levels are not changed. (c and d) Neonatal wmOPCs were differentiated for three days in the presence of ASTR‐conditioned medium (ACM) from untreated and cytokine (mixture)‐treated gmASTRs or wmASTRs. Myelin basic protein (MBP) immunocytochemistry is performed to assess differentiation (c, % MBP‐positive cells of DAPI‐stained cells) and myelin membrane formation (d, % myelin membranes formed by MBP‐positive cells; *n* = 3 independent cell culture preparations). Note that myelin membrane formation upon exposure to gmACM, but not to wmACM, is substantially reduced upon cytokine treatment of gmASTRs (*p* = .139, not significant). (e–h) mRNA levels of *Srebf2* (e), *Fdft1* (f), *Srebf1c* (g) or *Fasn* (h) in gmASTRs and wmASTRs that were either untreated (ctrl) or treated with a mixture of IL1β, IFNγ and TNFα for 24 hr (qPCR analysis of *n* = 3–4 independent cell culture preparations). Note that exposure to the pro‐inflammatory cytokine mix decreases transcripts for *Srebf1c* in wmASTRs and gmASTRs (wmASTRs *p* = .048, gmASTRs *p* = .008) and for *Fasn* in gmASTRs (*p* < .001). Bars represent relative means compared to exposure to control wmACM or gmACM (c and d) or untreated (ctrl) gmASTRs or wmASTRs (e–h), which are set to 1 in each independent experiment. Error bars represent standard error of the mean (*SEM*). Statistical analyses are performed using column statistics with a one‐sample *t*‐test (*p < .05, **p < .01, ***p < .001) to test for differences with control wmACM or gmACM (c and d) or untreated (ctrl) gmASTRs or wmASTRs (e–h). Absolute values of OPC differentiation with control wmACM are 21.8 ± 8.9% and with control gmACM 30.0 ± 8.3%. Absolute values of myelin membrane formation with control wmACM are 59.7 ± 8.3% and with control gmACM 70.5 ± 12.0%

MS is characterized by chronic inflammation (Chang et al., 2012; Compston & Coles, 2008; Nair et al., 2008; Park et al., 2019; Peterson, Bö, Mörk, Chang, & Trapp, 2001), and as ASTRs respond to inflammatory mediators, the effect of pro‐inflammatory cytokines and TLR agonists on cholesterol efflux was assessed. To this end, gmASTRs and wmASTRs were exposed either to a mixture of pro‐inflammatory cytokines (IFNγ, IL1β, and TNFα), Poly(I:C) or LPS for 24 hr. Poly(I:C) and LPS are agonists for TLR3 and TLR4, respectively. Cholesterol efflux from LPS‐treated gmASTRs was significantly reduced (Figure 3a, *p* = .033). Moreover, cholesterol efflux from cytokine‐treated gmASTRs and wmASTRs was also reduced by 35%–45% compared to the respective untreated ASTRs (Figure 3a, wm + cytokines *p* = .001; gm + cytokines *p*=<0.001). For all ASTR treatments, intracellular levels of cholesterol were not significantly changed compared to the levels in untreated ASTRs (Figure 3b). Exposure to ACM obtained from cytokine‐treated gmASTRs reproducibly, but not significantly reduced myelin membrane formation by wmOLGs (Figure 3d, grey bars). In contrast, the percentage of MBP‐positive cells was not altered compared to exposure to untreated control gmACM (Figure 3c, grey bars). Exposure to ACM obtained from cytokine‐treated wmASTRs did not alter wmOPC differentiation and myelin membrane formation (Figure 3c and d, white bars). To determine whether the cytokines interfered with cholesterol biosynthesis, the mRNA levels of *Srebf2* and *Fdft1* were examined. *Fdft1*is a gene encoding for squalene synthase (SQS), which is the first enzyme in the committed cholesterol biosynthesis pathway (Figure 2a). In contrast to previous findings (Memon et al., 1997; Park et al., 2016), neither *Srebf2* (Figure 3e) nor *Fdft1* (Figure 3f) mRNA levels were decreased in cytokine‐treated gmASTRs and wmASTRs. Instead, transcripts of both *Fdft1* and *Srebf2* seemed to increase in cytokine‐treated gmASTRs and wmASTRs, indicating a potential compensatory mechanism for the decreased cholesterol efflux from both types of ASTRs. In line with such a negative feedback loop is that *Srebf1c* mRNA levels were significantly decreased in cytokine‐treated wmASTRs (Figure 3g, *p* = .048) and gmASTRs (Figure 3g, *p* = .008). *Srebf1c* encodes for SREBP‐1c, the major transcription factor involved in unsaturated fatty acid synthesis (Shimano & Sato, 2017) (Figure 2a). Furthermore, mRNA levels of *Fasn*, encoding for the enzyme fatty acid synthase (Figure 2a), were significantly reduced in cytokine‐treated gmASTRs but not cytokine‐treated wmASTRs (Figure 3h, *p* < .001). These findings indicate that unsaturated fatty acid production and secretion may be reduced in cytokine‐treated ASTRs as well and thus may affect ASTR‐mediated modulation of myelin membrane formation. Hence, the cytokine‐induced decrease in cholesterol efflux from gmASTRs correlated with a decrease in ASTR‐mediated modulation of myelin membrane formation, as well as with a decrease in mRNA levels of genes encoding for a transcription factor and enzyme involved in unsaturated fatty acid synthesis.

### 3.4 Pro‐inflammatory cytokines inhibit cholesterol efflux from astrocytes via an ABCA1‐dependent pathway

**FIGURE 4:**
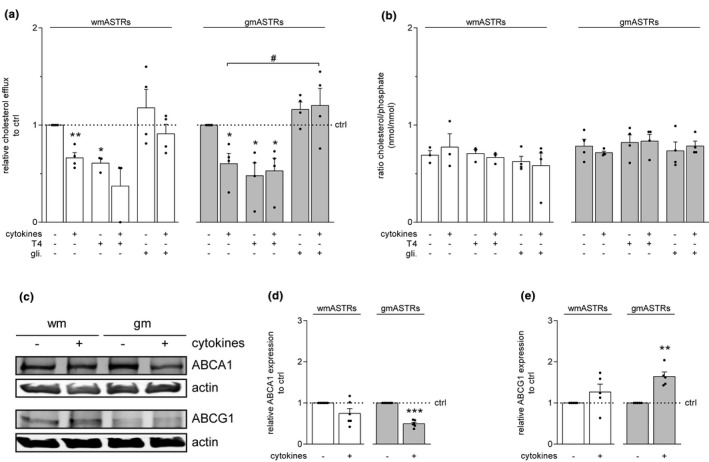
Pro‐inflammatory cytokines inhibit cholesterol efflux from astrocytes via an ABCA1‐dependent pathway. Primary neonatal grey matter (gm) and white matter (wm) astrocytes (ASTRs) were either untreated (ctrl) or treated for 24 hr with a mixture of pro‐inflammatory cytokines consisting of IL1β (1 ng/ml), IFNγ (500 U/mL) and TNFα (10 ng/ml) in the absence or presence of ABCA1 inhibitor glibenclamide (gli; 0.1 mM) or ABCG1 inhibitor thyroxine (T4; 50 µM). Inhibitors which were added to the cells one hour prior to addition of cytokines. (a,b) Cholesterol assays on gmASTRs and wmASTRs. Relative cholesterol efflux is shown in (a) and intracellular cholesterol levels, shown as intracellular cholesterol/phosphate ratios, in (b) (*n* = 3–4 independent cell culture preparations). Note that glibenclamide counteracts the cytokine‐induced reduction in cholesterol efflux from both wmASTRs and gmASTRs. (c–e) Western blot analyses of cholesterol transporters cholesterol transporters ABCA1 and ABCG1. Actin served as a loading control. Representative blots are shown in (c) and quantification for ABCA1 in (d) and for ABCG1 in (e) (*n* = 5–6 independent cell culture preparations). Note that exposure to cytokines decreases ABCA1 expression (*p* < .001) and increases ABCG1 expression (*p* = .004) in gmASTRs. Bars represent absolute values (b) or relative means (c,d,e) compared to control gmASTRs or wmASTRs, which are set to 1 in each independent experiment. Error bars represent standard error of the mean (*SEM*). Statistical analyses are performed using column statistics with a one‐sample *t*‐test (*p < .05, **p < .01, ***p < .001) to test for differences between control gmASTRs or wmASTRs. A one‐way ANOVA with a Šidák multiple comparisons post‐test (#*p* < .05) is used to test for differences between different treatments

Cholesterol is transported via passive transfer over the plasma membrane as well as via facilitated transport (Chen, Zhang, Kusumo, Costa, & Guizzetti, 2013). The main transporters of cholesterol in ASTRs are ATP‐binding cassette transporters A1 (ABCA1) and G1 (ABCG1) (Chen et al., 2013). To assess whether ABCA1 and/or ABCG1 contributed to the cytokine‐induced decrease in cholesterol efflux from ASTRs, the ABCA1 inhibitor glibenclamide (Terao et al., 2011) or the ABCG1 inhibitor thyroxine (T4) (Cserepes et al., 2004), were added one hour prior to treatment with the mixture of cytokines. T4, but not glibenclamide, significantly inhibited cholesterol efflux from untreated gmASTRs and wmASTRs by 40%–50% (Figure 4a, wm + glibencalamide, *p* = .015; gm + T4, *p* = .029). This is in line with previous findings that ABCG1 is the main cholesterol transporter in ASTRs (Karten, 2006). In contrast, glibenclamide, but not T4, prevented the cytokine‐induced reduction in cholesterol efflux from gmASTRs (Figure 4a, gm + cytokines versus gm + cytokines +glibenclamide, *p* = .027), and reproducibly, but not significantly from wmASTRs (Figure 4a, *p* = .074). Of note, simultaneous treatment with both inhibitors was toxic to ASTRs. Western blot analysis revealed that a mixture of pro‐inflammatory cytokines significantly decreased ABCA1 expression in gmASTRs (Figure 4c,d, gm + cytokines *p* < .001). In contrast, ABCG1 expression was significantly higher in cytokine‐treated gmASTRs than in untreated gmASTRs (Figure 4c and e, gm + cytokines *p* = .004). The effect of cytokines on ABCA1 and ABCG1 expression in wmASTRs was less pronounced (Figure 4c‐e). These findings demonstrate that although treatment with pro‐inflammatory cytokines decreased ABCA1 expression and increased ABCG1 expression, the cytokine‐induced reduction in cholesterol efflux from gmASTRs, and to a lesser extent from wmASTRs, depended on ABCA1 activity.

### 3.5 Inhibition of committed cholesterol biosynthesis in white matter astrocytes increases in vitro myelination

**FIGURE 5:**
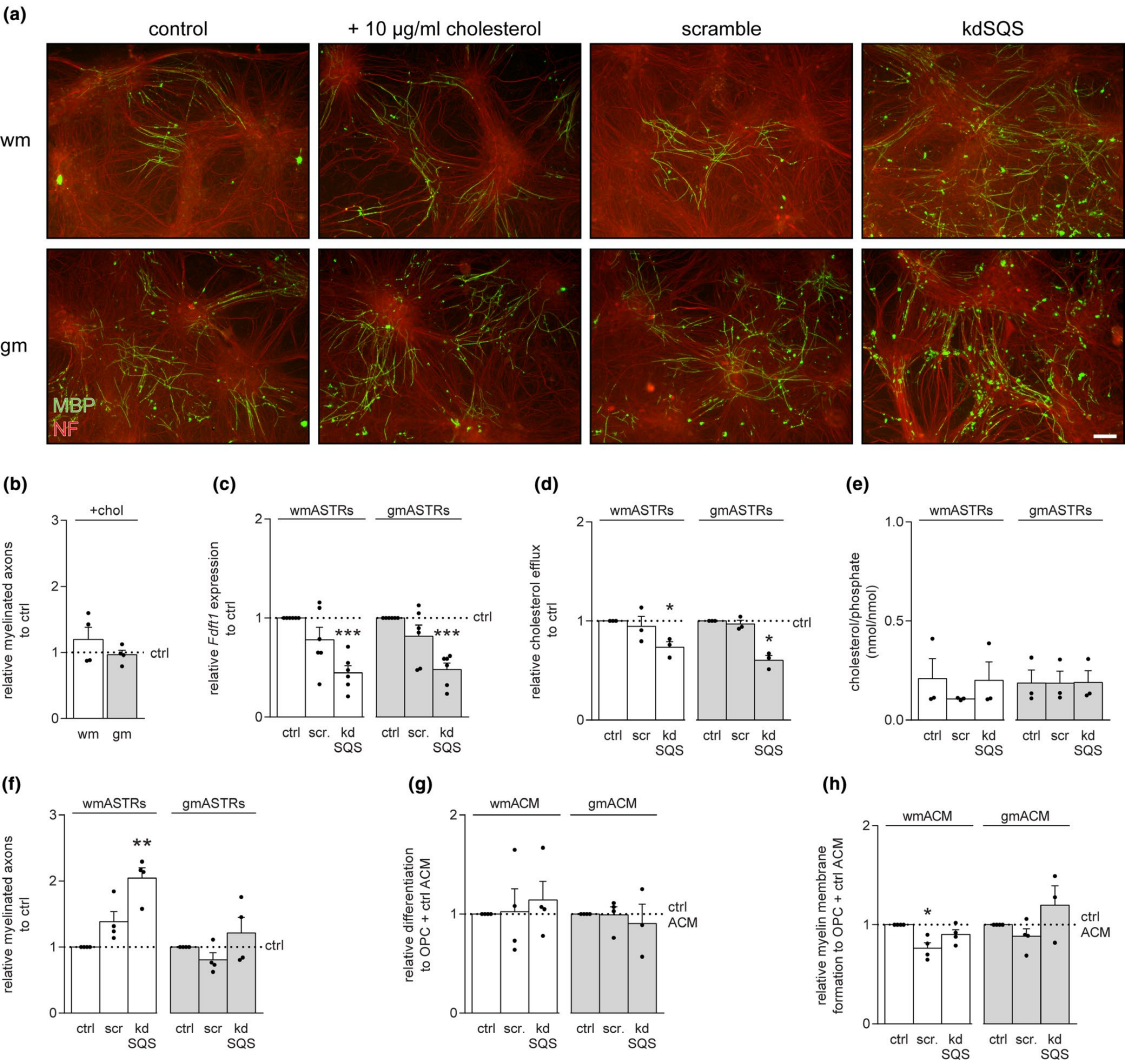
Inhibition of committed cholesterol biosynthesis in white matter astrocytes increases in vitro myelination. (a and b) In vitro myelinating cultures that depend on a feeding layer of astrocytes (ASTRs) are obtained from 15 days old rat embryo spinal cord cells and either left untreated (ctrl) or treated with 10 μg/ml cholesterol for the duration of the experiment. Cultures were stained for the myelin marker myelin basic protein (MBP, green) and the axonal marker neurofilament‐H (NF, red). Representative images are shown in (a) and quantification of the percentage of myelinated axons in (b) (*n* = 4 independent cell culture preparations). Note that addition of cholesterol does not affect in vitro myelination efficiency of both types of ASTRs. (a and c–e) A lentiviral shRNA knockdown (kd) of *Fdft1*, encoding for squalene synthase (SQS), was established in grey matter (gm)ASTRs and in white matter (wm)ASTRs (kdSQS). A shRNA scrambled (scr) construct was used as negative control. mRNA levels of *Fdft1* mRNA levels in control (ctrl), scr and kdSQS gmASTRs and wmASTRs is shown in (c) (qPCR analysis, *n* = 6 independent cell culture preparations), cholesterol efflux in (d) and intracellular cholesterol levels, shown as intracellular cholesterol/phosphate ratios, in (e) (*n* = 4 independent culture preparations). Note that *Fdft1* mRNA levels (wmASTRs and gmASTRs *p* < .001) and cholesterol efflux (wmASTRs *p* = .041 and gmASTRs *p* = .014) are decreased in kdSQS ASTRs. (a and f) In vitro myelinating cultures on a feeding layer of ctrl, scr, and kdSQS gmASTRs and wmASTRs. Representative images are shown in (a) and quantification of the percentage of myelinated axons in (f) (*n* = 4 independent cell culture preparations). (g) Neonatal wmOPCs were differentiated for three days in the presence of ASTR‐conditioned medium (ACM) from control, scr and kdSQS gmASTRs or wmASTRs. MBP immunocytochemistry is performed to assess differentiation (% MBP‐positive cells of DAPI‐stained cells) and myelin membrane formation (% myelin membranes formed by MBP‐positive cells) (*n* = 4 independent independent cell culture preparations). Bars represent relative means compared to control wmASTRs or gmASTRs (b), which are set to 1 in each independent experiment. Error bars represent standard error of the mean (*SEM*). Statistical analyses are performed using column statistics with a one‐sample *t*‐test (*p < .05, **p < .01, ** *p* < .001) to test for differences between control wmASTRs or gmASTRs. Absolute values of the percentage of myelinated axons on a feeding layer of control ASTRs are 3.2 ± 0.9% for wmASTRs and 6.7 ± 1.5% for gmASTRs. Absolute values for control ACM of wmASTRs are 20.5 ± 5.4% for OPC differentiation and 56.1 ± 7.9% for myelin membrane formation. Absolute values for control ACM of gmASTRs are 23.7 ± 2.4% and 49.9 ± 9.1%, respectively. Scale bar is 10 µm

To examine whether the higher secretion of cholesterol by gmASTRs contribute to enhanced myelination, cholesterol was added to the in vitro myelinating cultures that depends on an ASTR feeding layer. Upon continuous cholesterol treatment, the percentage of myelinated axons did not increase on a feeding layer of wmASTRs or gmASTRs at the end point of myelination (Figure 5a and b). Thus, the percentage of myelinated axons on wmASTRs was still reduced compared to gmASTRs (wmASTRs 3.5 ± 0.6% vs. gmASTRs 6.4 ± 1.4% *p* = .048). This indicates that the addition of cholesterol did not compensate for the lower secretion of cholesterol by wmASTRs or, alternatively, that an effect of inhibitory wmASTRs‐derived factors remained dominant. To test whether cholesterol may be taken up by other cell types and is thus not sufficiently supplied to OLGs, committed cholesterol biosynthesis was inhibited in both types of ASTRs via a shRNA knockdown (kd) of *Fdft1* (Figure 2a, kdSQS). As a negative control a scrambled shRNA construct was used. qPCR analysis revealed a 60%–65% decrease in *Fdft1* mRNA levels for both wmASTRs and gmASTRs (Figure 5c, wm kdSQS *p* < .001, gm kdSQS *p* < .001). In accordance, the cholesterol efflux from kdSQS ASTRs was reduced by 25%–40% compared to control and scrambled shRNA‐transduced ASTRs (Figure 5d wm, kdSQS *p* = .041, gm kdSQS *p* = .014). Moreover the reduction in cholesterol efflux was more prominent in gmASTRs than in wmASTRs. Intracellular cholesterol levels were unchanged (Figure 5e). Against our expectations, the percentage of myelinated axons did not decrease on a feeding layer of either kdSQS ASTRs. In fact, a two‐fold increase in myelination was observed on a feeding layer of kdSQS wmASTRs compared to a control wmASTR feeding layer (Figure 5a and f, kdSQS wmASTRs, *p* = .007). Furthermore, wmOPC differentiation and myelin membrane formation remained similar upon exposure to ACM from both kdSQS gmASTRs and kdSQS wmASTRs (Figure 5g and h), indicating that cholesterol is not the rate limiting factor and/or other factors may compensate for reduced cholesterol levels. Hence, surprisingly, inhibition of committed cholesterol biosynthesis downstream of the SQS substrate farnesyl‐pyrophosphate (farnesyl‐PP) in wmASTRs (Figure 2a) promoted, rather than inhibited, in vitro myelination. Upon kdSQS, pathways upstream of SQS‐mediated conversion of squalene to ultimately cholesterol, including non‐sterol isoprenoids and unsaturated fatty acid synthesis (Figure 2a), may become more active in kdSQS ASTRs and thereby compensate for the effect of reduced cholesterol levels, which was examined next.

### 3.6 Inhibition of committed cholesterol biosynthesis in white matter astrocytes increases *Sreb1fc* mRNA levels and substantially decreases pro‐IL1β secretion

**FIGURE 6:**
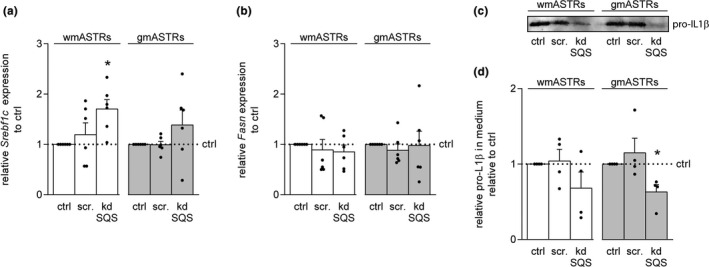
Inhibition of committed cholesterol biosynthesis in white matter astrocytes increased *Sreb1fc* RNA levels and substantially decreases pro‐IL1β secretion. (a–d) A lentiviral shRNA knockdown (kd) of *Fdft1*, encoding for squalene synthase (SQS) was established in grey matter (gm)ASTRs and in white matter (wm)ASTRs (kdSQS). A shRNA scrambled (scr) construct was used as negative control. mRNA levels of *Srebf1c* and *Fasn* on control (ctrl), scrambled (scr), and kdSQS in gmASTRs and wmASTRS are shown in (a) and (b), respectively (qPCR analysis, *n* = 6 independent cell culture preparations). Representative Western blots of pro‐IL1β levels in 80 µl ASTR‐conditioned medium (ACM) are shown in (c) and quantification in (d) (*n* = 4 independent cell culture preparations). Note that the kdSQS in wmASTRs increases *Srebf1c* mRNA levels in wmASTRs (*p* = .013), but not in gmASTRs, and that pro‐IL1β secretion is substantially reduced in ACM of kdSQS wmASTRs (not significant) and significantly reduced in kdSQS gmASTRs (*p* = .032). Bars represent relative means to control gmASTRs or wmASTRs, which are set to 1 in each independent experiment. Error bars represent standard error of the mean (*SEM*). Statistical analyses are performed using column statistics with a one‐sample *t*‐test (*p < .05) to test for differences between untreated control gmASTRs or wmASTRs

To examine whether an inhibition of cholesterol synthesis downstream of farnesyl‐PP results in an increase in fatty acid synthesis (Figure 2a), the mRNA levels of *Srebf1c* were examined by qPCR. An increase in *Srebf1c* transcripts was observed in kdSQS wmASTRs compared to control wmASTRs (Figure 6a, *p* = .013), indicating that unsaturated fatty acid production may be increased in kdSQS wmASTRs. mRNA levels of *Srebf1c* were similar in control gmASTRs and kdSQS gmASTRs (Figure 6a). Transcripts of *Fasn* were not significantly changed in kdSQS wmASTRs and kdSQS gmASTRs (Figure 6b). Like committed cholesterol biosynthesis, the generation of non‐sterol isoprenoids is also a downstream pathway of farnesyl‐PP (Veluthakal, Arora, Goalstone, Kowluru, & Kowluru, 2016) (Figure 2a). Of note, non‐sterol isoprenoids are hydrocarbon chains used to anchor several signaling proteins to cell membranes, a process called isoprenylation (Caraglia et al., 2005; Lindholm & Nilsson, 2007). Interestingly, when blocking cholesterol biosynthesis pathway upstream of farnesyl‐PP with the HMG‐CoA reductase inhibitor simvastatin (Figure 2a), the secretion of several cytokines, including IL1β, is increased by inhibition of isoprenylation‐dependent signaling pathways (Lindholm & Nilsson, 2007; Massonnet et al., 2009). In line with these observations, pro‐IL1β levels in ACM of kdSQS gmASTRs were reduced compared to pro‐IL1β levels in ACM of control gmASTRs (Figure 6c,d, *p* = .032). Similarly, pro‐IL1β levels were substantially, but not significantly, reduced in ACM of kdSQS wmASTRs (Figure 6c and d). Notably, active IL1β was not detected in the medium, probably because of its short half‐life after secretion (Kudo, Mizuno, Hirai, & Shimizu, 1990). Hence, impairing committed cholesterol biosynthesis in wmASTRs enhanced in vitro myelination, likely by increased unsaturated fatty acid biosynthesis, as well as non‐sterol isoprenoid biosynthesis.

## 4 DISCUSSION

It is known that remyelination is not only more efficient in GM MS lesions than in WM MS lesions (Chang et al., 2012; Strijbis et al., 2017), but also faster in GM than in WM upon toxin‐induced demyelination (Bai et al., 2016). ASTRs are important players in the remyelination process, and malfunction of ASTR signaling contributes to remyelination failure in WM MS lesions (Gutowski, Newcombe, & Cuzner, 1999; Harlow & Macklin, 2014; Lau et al., 2012; Lundgaard, Osório, Kress, Sanggaard, & Nedergaard, 2014; Stoffels et al., 2013). Therefore, we hypothesized that in addition to wmASTRs being more detrimental, gmASTRs may be more supportive for remyelination by supplying more cholesterol to developing OLGs. Our findings demonstrate that cholesterol efflux from gmASTRs was higher than from wmASTRs. This increase in cholesterol secretion correlated with enhanced wmOPC differentiation upon exposure to gmACM, but not wmACM, and a more supportive role of gmASTRs than wmASTRs in in vitro myelination. Although inhibition of committed cholesterol synthesis by a knockdown of SQS reduced cholesterol efflux from ASTRs, in vitro myelination remained similar on a feeding layer of kdSQS gmASTRs. Moreover*,* in vitro myelination was enhanced on a feeding layer of kdSQS wmASTRs. Hence, cholesterol supply by ASTRs is not rate‐limiting for in vitro myelination, and more importantly, our findings demonstrated that specific blocking of cholesterol biosynthesis in wmASTRs was in fact beneficial for in vitro myelination. This may open new ASTRs‐based therapeutic strategies that aim to promote remyelination in WM MS lesions.

Against our expectations, in vitro myelination was enhanced upon reduction of committed cholesterol biosynthesis by a knockdown of SQS in wmASTRs, indicating that other factors likely masked the effect of reduced levels of ASTR‐derived cholesterol. Cholesterol, non‐sterol isoprenoids and unsaturated fatty acids have acetyl‐CoA as a common precursor making their synthesis intertwined and tightly coordinated by the SREBP family of transcription factors (Figure 2a). Our findings revealed that transcripts of *Sreb1fc*, which activates unsaturated fatty acid biosynthesis, were increased in kdSQS wmASTRs but not in kdSQS gmASTRs compared to control ASTRs. As addition of polyunsaturated fatty acids promotes OPC differentiation (van Meeteren, Baron, Beermann, Dijkstra, & van Tol, 2006), increased unsaturated fatty acid biosynthesis may contribute the observed enhanced in vitro myelination upon a feeding layer of kdSQS wmASTRs. In addition, a previous study showed that upon restricting synthesis of both cholesterol and non‐sterol isoprenoids in macrophages by the HMG‐CoA reductase inhibitor simvastatin, the secretion of IL1β and IL8 increases, while the secretion of TNFα decreases, through an isoprenylation‐dependent mechanism (Lindholm & Nilsson, 2007). In line with these findings, upon specific blocking of cholesterol biosynthesis in ASTRs, as established here with kdSQS, more substrate likely became available for non‐sterol isoprenoid synthesis, which may explain the reduced pro‐IL1β secretion from kdSQS ASTRs. Although findings on IL1β affecting OPC differentiation are conflicting (Vela, Molina‐Holgado, Arévalo‐Martín, Almazán, & Guaza, 2002; Xie et al., 2016), IL1β inhibits wmOPC differentiation in vivo (Xie et al., 2016). This indicates that statin‐mediated inhibition of remyelination in the corpus callosum (Klopfleisch et al., 2008; Miron et al., 2009) may also be in part unrelated to cholesterol production and linked to altered ASTR reactivity and secretome as a result of reduced non‐sterol isoprenoid synthesis. Indeed, statins induce an astroglia and microglia response in the demyelinated corpus callosum, which hints to alterations in the inflammatory environment (Miron et al., 2009). Hence, both enhanced unsaturated fatty acid synthesis and non‐sterol isoprenoid biosynthesis in ASTRs, the latter likely resulting in increased isoprenylation and the subsequent reduced secretion of ASTR‐derived cytokines that affect OPC maturation, may contribute to enhanced *in* vitro myelination in the presence of kdSQS wmASTRs despite reduced cholesterol levels.

In line with the present findings, differences between GM and WM myelination upon inhibition of lipid biosynthesis in ASTRs has been described. ASTR specific deletion of SREBP‐cleavage‐activating protein (SCAP) that post‐transcriptionally activates SREBPs (Shimano & Sato, 2017) results in a decrease of WM volume of approximately 60%, while the reduction in GM was only 10% (Camargo et al., 2017; Lindholm & Nilsson, 2007; Massonnet et al., 2009; Xie et al., 2016). Whether this is because of the lower amount of myelin present in GM or that wmOPC differentiation, as observed in the present study, is more affected by the reduced synthesis of ASTR‐derived lipids is not studied yet. Similarly, hypomyelination in WM but not in GM may partially relate to reduced biosynthesis of non‐sterol isoprenoids. As a result, increased secretion of several ASTR‐derived cytokines may delay and/or reduce wmOPC differentiation and myelination (Camargo et al., 2017; Lindholm & Nilsson, 2007; Massonnet et al., 2009; Xie et al., 2016). In fact, wmOPC differentiation may depend more on secreted factors from ASTRs than gmOPC differentiation, including cholesterol, and wmOPCs are more susceptible to the effect of pro‐inflammatory cytokines than gmOPCs (Lentferink et al., 2018). Of relevance, upon SQS deletion in OLGs, which makes OLGs dependent on cholesterol supply by other cells, myelin appears of normal thickness in GM, whereas it is very thin in WM (Saher et al., 2005). Although it cannot be excluded that this regional difference in the involvement of cholesterol in myelin membrane growth depends on an intrinsic difference between gmOPCs and wmOPCs, it is likely that a higher cholesterol efflux from gmASTRs, as is suggested (Saher et al., 2005) and shown here, is involved.

Our findings indicate that cholesterol efflux from gmASTRs and wmASTRs is regulated by inflammatory mediators. While TLR4 agonist LPS more potently decreased cholesterol efflux from gmASTRs than from wmASTRs, a mixture of pro‐inflammatory cytokines decreased cholesterol efflux from both ASTR types to a similar extent. However, only secreted factors from cytokine‐treated gmASTRs reproducibly decreased myelin membrane formation. Hence, the remaining cholesterol levels were sufficient and not rate‐limiting for OPC differentiation in vitro. Remarkably, *Srebf2* and *Fdft1* mRNA levels were not reduced by cytokine treatment of gmASTRs and wmASTRs, indicating that cytokines may interfere with the expression and/or function of cholesterol transporters. Indeed, ABCA1 expression and cholesterol efflux via ABCA1 were reduced in cytokine‐treated gmASTRs. Besides, the increased ABCG1 expression in cytokine‐treated gmASTRs did not enhance cholesterol efflux, which is consistent with a previous study demonstrating that an increased expression of ABCG1 does not enhance cholesterol efflux per se (Terao et al., 2011). Of note, treatment with an ABCA1 agonist increased gene expression of enzymes involved in cholesterol biosynthesis in ASTRs, and improved clinical outcome in experimental autoimmune encephalomyelitis (Itoh et al., 2018). Our results further revealed that exposure to a mixture of pro‐inflammatory cytokines reduced mRNA levels of *Srebf1c* in gmASTRs and wmASTRs. In addition, the mRNA expression of *Fasn* was reduced in cytokine‐treated gmASTRs, but not in cytokine‐treated wmASTRs. Hence, exposure to pro‐inflammatory cytokines likely interfered with both cholesterol efflux and unsaturated fatty acid synthesis. As inflammatory activity is lower in GM MS lesions than in WM MS lesions (Chang et al., 2012; Clarner et al., 2012; Prins, Schul, Geurts, Valk, & van der, Drukarch B., Dam A.‐M. van, 2015; Strijbis et al., 2017), the cytokine‐induced decrease in cholesterol efflux from ASTRs may be of more relevance for the reduced remyelination efficiency in WM MS lesions.

Taken together, the present study showed a positive correlation between cholesterol secretion and OPC differentiation as well as in vitro myelination. However, using the kdSQS in vitro myelination model presented in this study, it could not be confirmed whether the higher levels of secreted cholesterol by gmASTRs play a role in the enhanced myelination efficiency by gmASTRs compared to wmASTRs. Intriguingly, using this kdSQS model, we identified an under investigated role of non‐sterol isoprenoids. Non‐sterol isoprenoids share a common precursor with the cholesterol and unsaturated fatty acid biosynthesis pathway and regulate the secretion of cytokines and in this way contributes to ASTR‐mediated modulation of OPC differentiation and myelin membrane formation. Of interest, from a translational perspective, interfering with lipid metabolism in ASTRs, that is, increasing non‐sterol isoprenoid synthesis without interfering with cholesterol biosynthesis, may be a novel strategy to reduce the secretion of ASTR‐derived pro‐inflammatory cytokines and to promote remyelination in WM MS lesions. Indeed, a lipid‐enriched diet is sufficient to rescue hypomyelination and neurological deficits in conditional SCAP^‐/‐^ ASTR mice (Camargo et al., 2012, 2017) and addition of dietary cholesterol accelerates WM remyelination upon toxin‐induced demyelination (Berghoff et al., 2017). Hence, an increased lipid supply is beneficial for remyelination, and may act on SREBPs in ASTRs, thereby likely modulating non‐sterol isoprenoid synthesis as well.

## CONFLICTS OF INTEREST

The authors declare that they have no conflicts of interest.

## AUTHOR CONTRIBUTIONS

I.L.W. and W.B. designed the project. W.B. supervised the study. I.L.W., J.K., and J.C.dJ performed the experiments and acquired the data. I.L.W. produced the figures and carried out the statistical analysis. I.L.W. wrote the draft manuscript text, J.K. edited the manuscript text, and W.B. critically reviewed and revised the manuscript text. All authors read and approved the manuscript.
